# Tribological Characterization of Carbon Fibre Reinforced Polymer (CFRP) Sliding Against Ti and Al Alloy Counterbodies for Aerospace Applications

**DOI:** 10.3390/ma18184296

**Published:** 2025-09-13

**Authors:** Luís Vilhena, Sharjeel Ahmed Khan, André Garcia, Amílcar Ramalho

**Affiliations:** CEMMPRE—Centre for Mechanical Engineering, Materials and Processes, University of Coimbra, Rua Luís Reis Santos, 3030-788 Coimbra, Portugal; sharjeel.focus@gmail.com (S.A.K.); andreneutel00@gmail.com (A.G.); amilcar.ramalho@dem.uc.pt (A.R.)

**Keywords:** carbon fibre reinforced polymer (CFRP), friction, wear, aerospace applications

## Abstract

Carbon fibre reinforced polymer (CFRP) is a composite material known for its light weight and exceptional durability, composed of carbon fibres within a polymer matrix. Despite its high cost, CFRP is favoured for its outstanding strength-to-weight ratio and rigidity. It is widely used in the aerospace industry and ship superstructures, among others. These components often rub against different materials in various structural and mechanical assemblies. These interactions typically occur where metallic fasteners, bearings, hinges, and sliding components interface with CFRP parts causing, for example, fretting wear. The main novelty of the present study consists of a systematic comparison of titanium (Ti6Al4V) and aluminium (AA2024-T6) alloy spheres under identical test conditions, evaluating how each material interacts with different CFRP configurations. CFRP was tested against titanium and aluminium alloy spheres as counterbodies under reciprocating sliding conditions. Different contact conditions (applied loads) were used for tribotests. The wear volume and coefficient of friction were determined, as well as the wear mechanisms. Different analytical techniques were employed, such as profilometry, optical microscopy (OM), and scanning electron microscopy (SEM/EDS), to characterise the wear tracks. It was possible to determine the coefficient of friction as well as the wear rate on both CFRP specimens and their respective counterbodies. It was found that the coefficient of friction (CoF) depends on load, fibre orientation, and counterbody material, ranging from 0.14 to 0.29. The lowest wear rate coefficient was observed for CFRP sliding against titanium alloy in the layer configuration, at 1.48 × 10^−13^ mm^3^/N·m. In contrast, aluminium alloy counterbodies experienced significantly higher wear, with a maximum wear rate of 6.88 × 10^−5^ mm^3^/N·m. Wear volume increased with load across all conditions and was highest for the CFRP cross-section against aluminium alloy.

## 1. Introduction

Carbon fibre reinforced polymer (CFRP) is a composite material valued for its light weight, durability, and high strength-to-weight ratio. It consists of carbon fibres embedded in a polymer matrix, where the fibres provide superior strength, and the matrix binds them together. Typically, CFRP’s structure includes multiple fibre layers oriented at different angles, creating regions with varying strength and stiffness. These unique properties make CFRP ideal for use in aerospace, automotive, and civil engineering applications, where performance is crucial [1,2,3,4,5,6,7,8].

CFRP’s tensile properties, low weight, and low thermal and electrical conductivity make it particularly significant in the aerospace industry [9,10]. Another material, titanium, is also frequently used in aerospace due to its high strength-to-weight ratio and corrosion resistance [5]. Combining CFRP and titanium in CFRP-Ti stacks (used in components like fuselages and wing spars) results in a hybrid structure that offers enhanced performance [11]. However, machining these stacks, especially drilling, presents challenges due to the hard-to-machine nature of both materials, leading to issues such as tool wear and surface finish problems [12,13,14].

Several studies have explored the tribological behaviour of CFRP, focusing on the effects of factors like load, temperature, and fibre orientation on wear and friction, particularly under dry friction conditions and in contact with materials such as tungsten carbide, aluminium, or titanium. Liang and Wu [6,7] investigated CFRP’s wear mechanisms under varying loads, temperatures, and sliding speeds. They found that wear generally increased with load and temperature until reaching a certain point, after which it decreased, reflecting uncertainty about whether wear and friction scale predictably with load. Birleanu et al. [9] discovered that the use of lubricants significantly reduced the coefficient of friction (CoF) in CFRP, and higher loads led to a lower CoF. However, dry sliding conditions are more severe, but realistic for certain applications.

Fibre orientation plays a crucial role in determining tool wear during machining processes. For example, Nguyen et al. [15] demonstrated that different fibre orientations in CFRP affect tool wear during edge trimming, with 45° plies causing the most significant wear, while 0° plies caused the least. Hu et al. [10] examined the fretting behaviour of CFRP in contact with titanium alloys. They found that fibre orientation and temperature significantly influence the coefficient of friction (CoF) and wear, with different fibre orientations exhibiting distinct CoF values across varying temperatures. These facts create controversy around the generalizability of fibre orientation effects, particularly under different tribological conditions (fretting, reciprocating sliding, etc.).

Research has shown that CFRP-Ti interactions can offer performance advantages: Wang et al. [11] found that the carbon fibres in CFRP can reduce tool wear by removing titanium adhesion during machining, thereby extending tool life. Suo et al. [16] showed that higher fibre angles in CFRP-Ti bolted joints resulted in reduced wear and CoF, further demonstrating the importance of fibre orientation in composite-metal stacks.

Composite-metal stacks like CFRP-Al and CFRP-Ti are in high demand. However, the fretting and dynamic behaviour between these materials, particularly when connected with mechanical bolts near hinges or connectors, can degrade material properties due to high stress generation.

**Figure 1:**
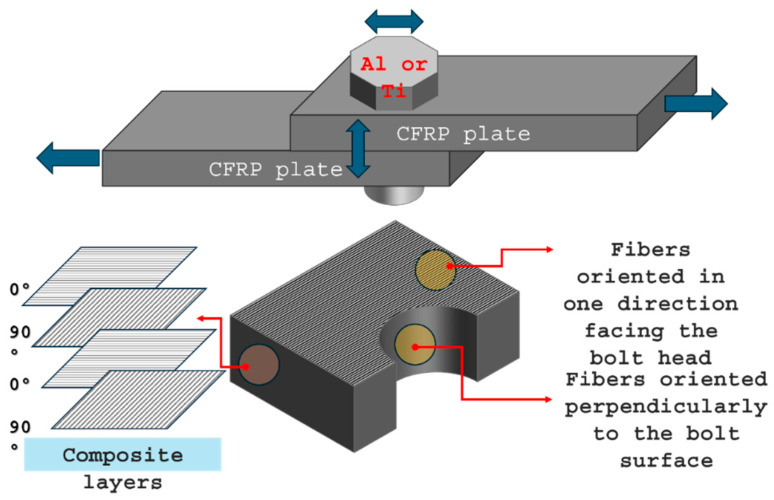
Schematic picture showing CFRP interaction with Ti and Al fasteners.

CFRP interacts with Ti and Al in fasteners, landing gear, wings, engines, and doors structures, as exemplified in the schematic picture of Figure 1. These interfaces pose tribological challenges like fretting, wear, and galvanic corrosion, requiring solutions such as coatings, lubricants, and isolators to improve longevity and performance.

This study seeks to address the shortage of systematic experimental research comparing the wear and friction performance of CFRP against titanium and aluminium alloys, particularly under reciprocating motion and varying fibre orientations. To fill this gap, we employ a custom-designed tribometer and conduct a detailed analysis of wear mechanisms from both microstructural and mechanical perspectives. A metal ball was subjected to reciprocating sliding motion against a CFRP specimen in a ball-on-flat configuration, investigating the influence of different contact conditions and fibre orientations on wear and friction behaviour.

## 2. Materials and Methods

### 2.1. Materials

CFRP specimens were manufactured using a Hexply^®^ 8552 (Hexcel, Stamford, CT, USA) prepreg arranged in a (0°, 90°) orientation consisting of multiple layers to achieve the desired thickness of 7.3 mm. Hexply^®^ 8552 is a toughened epoxy-based unidirectional carbon fibre pre-impregnated (prepreg), laid up in a [0°, 90°] cross-ply orientation for balanced stiffness. After stacking, the laminate is vacuum-bagged and cured in an autoclave at 5 bar and ~180 °C for 2 h. This process enhances resin flow, compacts the fibres, and minimizes void content for improved mechanical performance. The manufactured details can be found elsewhere [12], and the CFRP specifications can be seen in Table 1.

**Figure 2:**
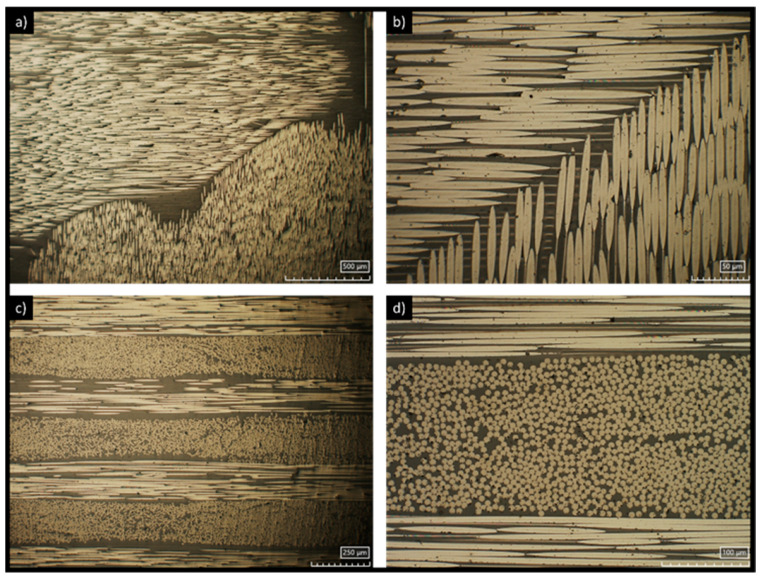
Micrographs for different magnifications obtained using Hirox equipment (Limonest, France), showing the CFRP layers (**a**,**b**) and respective cross-sections (**c**,**d**).

Figure 2 shows different micrographs of CFRP specimens used for the tribotests. The specimens were prepared for metallography by mounting them in acrylic resin. The top part (layer) and the cross-section (CS) were first polished with different grinding papers and diamond paste of 3 μm, and then examined using a 3D digital microscope, Hirox HR-5000 (Limonest, France). The layer part, represented in Figure 2a,b, shows the carbon fibres aligning in alternating orientations, with the first layer parallel to the length of the structure (0°) and the next layer perpendicular (90°). The cross-section, as shown in Figure 2c,d, displays the same carbon fibres, but in this case they are oriented in two directions simultaneously (bidirectional), with one set at 0° and the other at 90°.

During the present research work, various specimens were utilized with different combinations, constituting different tribopairs. The CFRP specimens with different fibre orientations (layers (CFRPLayer) and cross-sections (CFRPCS)) were tested against different spheres of titanium alloy (Ti6Al4V) and aluminium alloy (anodized aluminium 2024 with T6 heat treatment) with 5 mm radius, constituting four different tribopairs. The chemical composition of both titanium and aluminium alloy counterbodies can be seen, respectively, in Table 2 and Table 3.

The Vickers hardness was measured using an automatic tester Struers Duramin system (Champigny sur Marne, France). A 1 kg load (9.8 N) was applied for 15 s dwell time (HV1), yielding hardness values of 302 kgf/mm^2^ and 137 kgf/mm^2^ for titanium (Ti) and aluminium (Al) alloys, respectively. The arithmetic average roughness (Ra) was measured using a Mitutoyo Surftest SJ-500 device from Mitutoyo Corporation (Kanagawa, Japan). The Ra values obtained for the CFRP specimens in the layer and cross-section configurations were 1.11 µm and 1.33 µm, respectively. Regarding the tested counterbodies made of aluminium and titanium alloys, both had a Ra lower than 0.5 µm.

### 2.2. Methods

**Figure 3:**
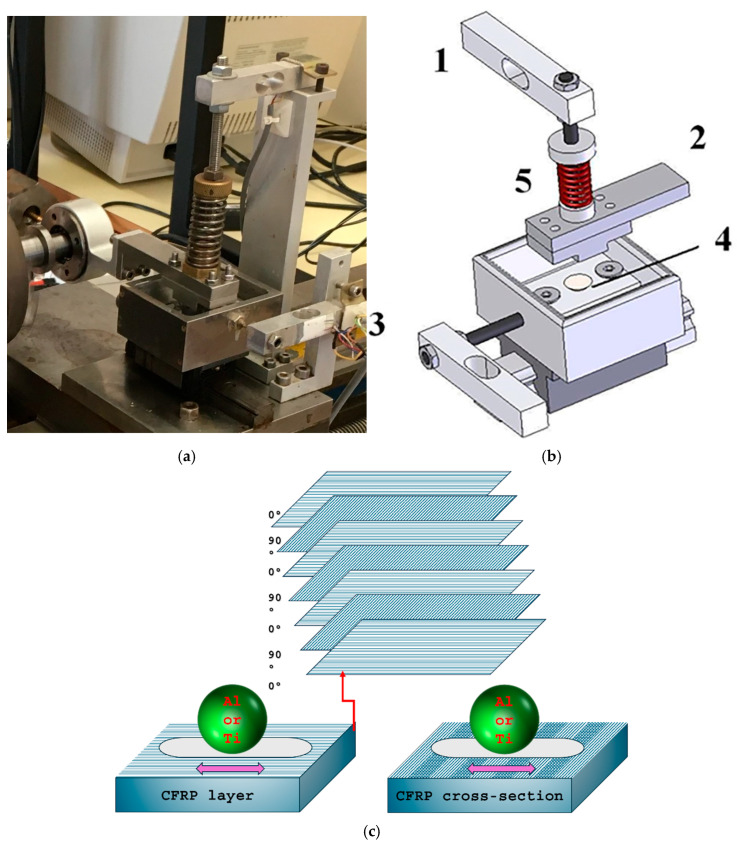
Reciprocating sliding tribometer used for tribological tests: (**a**) tribometer detail; (**b**) schematic picture showing the different components; (**c**) CFRP specimens with different fibre orientations (dimensions 20 mm × 20 mm × 7.3 mm).

During the tribotests, dry tests were conducted using a home made reciprocating sliding tribometer with ball-on-flat configuration. The ball was passed through a cyclic motion with a stroke length of 2 mm. CFRP specimens with different fibre orientations (layers (CFRPLayer) and cross-sections (CFRPCS)) were tested against titanium and aluminium alloy spheres, as shown in Figure 3c. A tribometer built and designed at our lab was used, as shown in Figure 3a. Figure 3b shows a diagram illustrating the reciprocating sliding wear test shown in Figure 3a. In this setup, the sphere that is made of aluminium and/or titanium alloy is attached to a moving stage (2) and maintains continuous contact with the horizontal surface of the CFRP stationary specimen (4). A spindle-spring (5) applies the normal load, which is measured by a normal load cell (1). Additionally, a stationary load cell (3) is used to balance the lower specimen, thereby measuring the friction force values throughout the test. The load cells are then connected to LabVIEW software (version spring 2020) on a computer to facilitate data acquisition and analysis. Three tribotests for the same contact conditions were performed, and then 2D profiles were obtained using the profilometer mentioned above and integrated using the trapezoidal rule to determine the average volume of each condition. The reduction in volume of the Ti and Al ball was determined using the following equation to determine the spherical cap (Equation (1)) [17].

(1) $$V=\left(\frac{\pi h}{6}\right)\left[\frac{3d^{2}}{4}+h^{2}\right]$$

where h = r − [r^2^ − d^2^/4]^1/2^ (height of material removed);

d = diameter of the wear scar;
r = ball radius.

In addition, the specific wear rate coefficient (k), measured in units of mm^3^/Nm, was determined using Archard’s wear equation, which states that the wear volume is directly proportional to the sliding distance (m) multiplied by the applied normal load (N) (severity). Table 4 presents the tribological test conditions for the four different tribopairs.

The load range was selected based on preliminary tests and prior studies that confirmed excessive deformation and wear of the softer aluminium alloy at higher loads. The 10–30 N range allowed for consistent, reproducible wear patterns in the Ti alloy group, while, for Al, loads above 20 N caused severe damage to both ball and composite, compromising measurement accuracy. The tribological tests were conducted under ambient laboratory conditions, with temperature and humidity monitored during the tests (T ≈ 22 °C, RH ≈ 45%).

Despite the anisotropic nature of CFRP, effective isotropic properties were used as a first-order approximation to determine the contact radius and maximum contact pressure based on Hertzian contact theory described elsewhere [18]. This approach provides a conservative estimate of local stress under normal loading conditions. The results are presented in Table 5. The mechanical properties used in the contact analysis were Ti6Al4V (E = 113 GPa, ν = 0.34) and AA2024-T3 (E = 73 GPa, ν = 0.33), as reported in [19]. For the CFRP, an effective Young’s modulus of 45 GPa and Poisson’s ratio of 0.30 were assumed based on [20] and representative data from [21].

## 3. Results and Discussion

### 3.1. Friction Behaviour

#### 3.1.1. CFRP Sliding Against Ti Alloy Sphere as Counterbody

**Figure 4:**
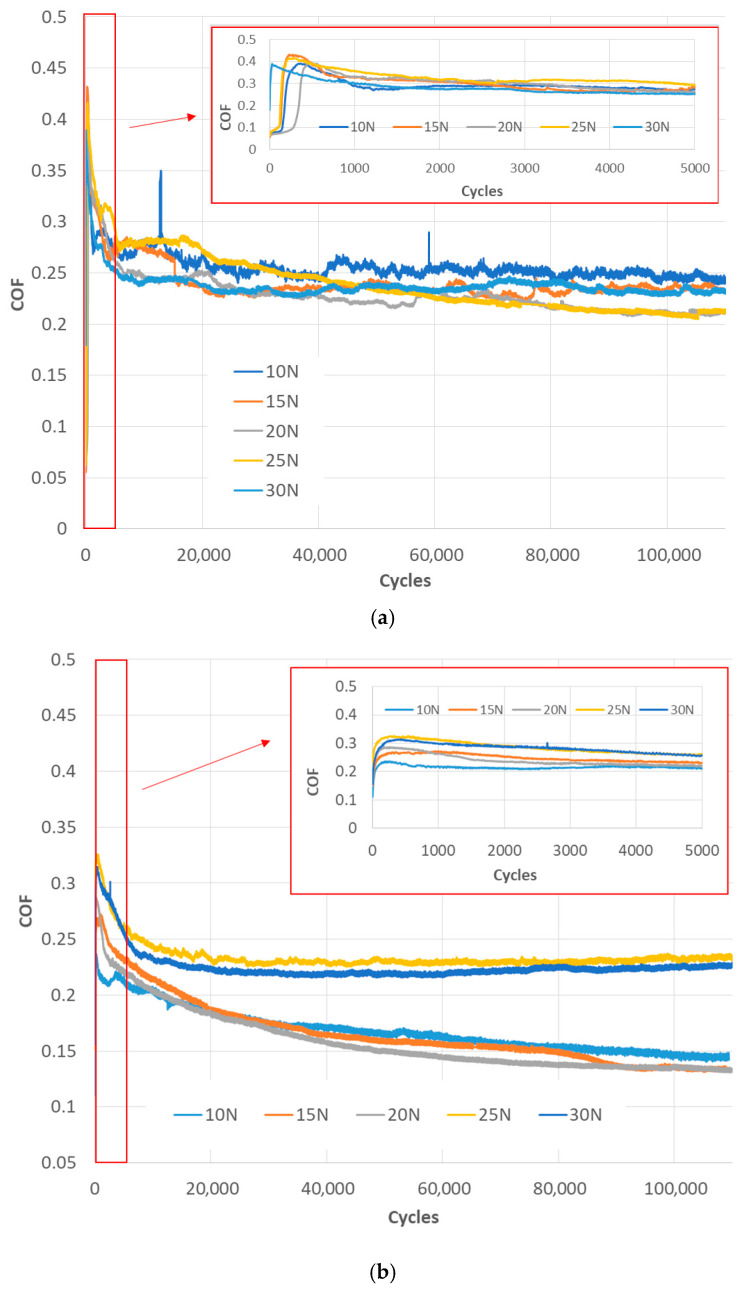
Evolution of the CoF with the number of cycles for CFRP specimens sliding against Ti alloy sphere under different applied loads (10, 15, 20, 25, and 30 N) for (**a**) CFRPLayer specimen; (**b**) CFRPCS specimen.

Upon analysis of Figure 4a, which depicts the coefficient of friction (CoF) as a function of the number of cycles for the tribosystem consisting of a CFRPLayer sliding against a Ti sphere for varying applied loads, several similarities emerge across all tests. At the outset of the tests, the coefficient of friction (CoF) is markedly elevated, reaching approximately 0.4. This contrasts with the subsequent phases, where the CoF exhibits a stabilising effect, reaching a value of approximately 0.25. This initial behaviour can be attributed to the initial running-in process, which is particularly pronounced in the mini-graph that extends up to 5000 cycles. The coefficient of friction (CoF) initially increases but then stabilises at approximately the same level after 600 cycles, a trend that is consistent across all tests.

Following the completion of the running-in process, the coefficient of friction (CoF) values observed across the tests exhibited a range of 0.2 to 0.25. Despite a 20 N discrepancy in the applied loads, the CoF values exhibit a narrow range of 0.05, indicating comparable performance. The highest coefficient of friction (CoF) value of 0.25 is observed in the 10 N test, which corresponds to the lowest applied force. In contrast, the 25 N test demonstrates the lowest coefficient of friction (CoF) value, exhibiting the most pronounced decrease in CoF from the initial to the final stages of the test.

A detailed examination of Figure 4b, which illustrates the evolution of the coefficient of friction (CoF) with the number of cycles for the CFRPCS specimen sliding against a titanium alloy sphere under varying applied loads, reveals the presence of two discernible trends. As with the CFRPLayer, all tests initially exhibit a markedly elevated coefficient of friction (CoF) of approximately 0.33, which subsequently stabilises to a value of around 0.25 or 0.15 during the subsequent phases of the test. This initial spike in CoF is attributed to the running-in process, as clearly illustrated in the accompanying graph, which covers up to 5000 cycles. Following approximately 1000 cycles, the CoF value stabilises, exhibiting no significant increase or decrease.

Once the running-in process is complete, the CoF values in the tests settle into two distinct ranges. The initial three tests, conducted at 10, 15, and 20 N, demonstrate strikingly similar CoF values, approximately 0.15. In contrast, the final two tests with higher loads, 25 and 30 N, demonstrate a higher coefficient of friction (CoF) of approximately 0.24. The 25 N test exhibits the highest CoF, while the 20 N and 15 N tests demonstrate the lowest COF.

**Figure 5:**
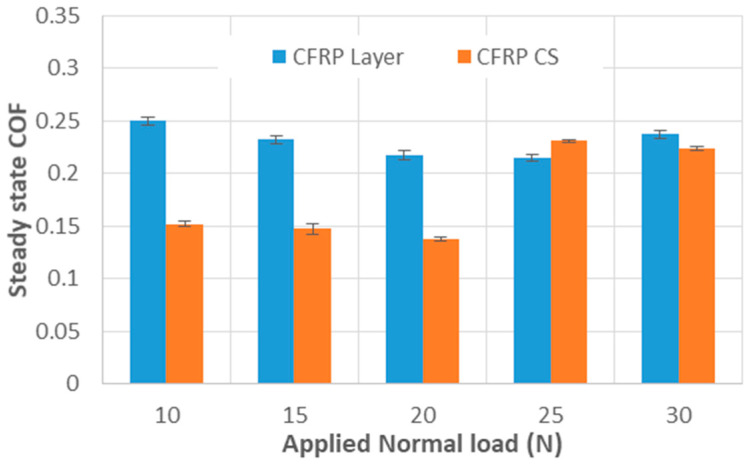
Steady-state CoF for CFRP (CFRPLayer and CFRPCS) sliding against titanium alloy sphere under different applied loads—the steady-state region, typically from cycle 1000 to the end, was used to compute the mean CoF and standard deviation (The coefficient of variation ranged from a minimum of 0.66% to a maximum of 3.35%).

To compare the CoF values under different applied loads, the average CoF and standard deviation were calculated, as shown in Figure 5. For the CFRPLayer, the figure clearly indicates that the test conducted at a 25 N applied load exhibits the lowest CoF, approximately 0.22, while the test conducted at a 10 N applied load shows the highest CoF of 0.25.

For the CFRPCS, two distinct CoF values are evident: approximately 0.15 for normal loads up to 20 N and around 0.23 for the highest two loads. Moreover, the lowest CoF value, 0.14, is associated with a load of 20 N, while the highest CoF value, 0.23, corresponds to a load of 25 N.

#### 3.1.2. CFRP Sliding Against Al Alloy Sphere as Counterbody

**Figure 6:**
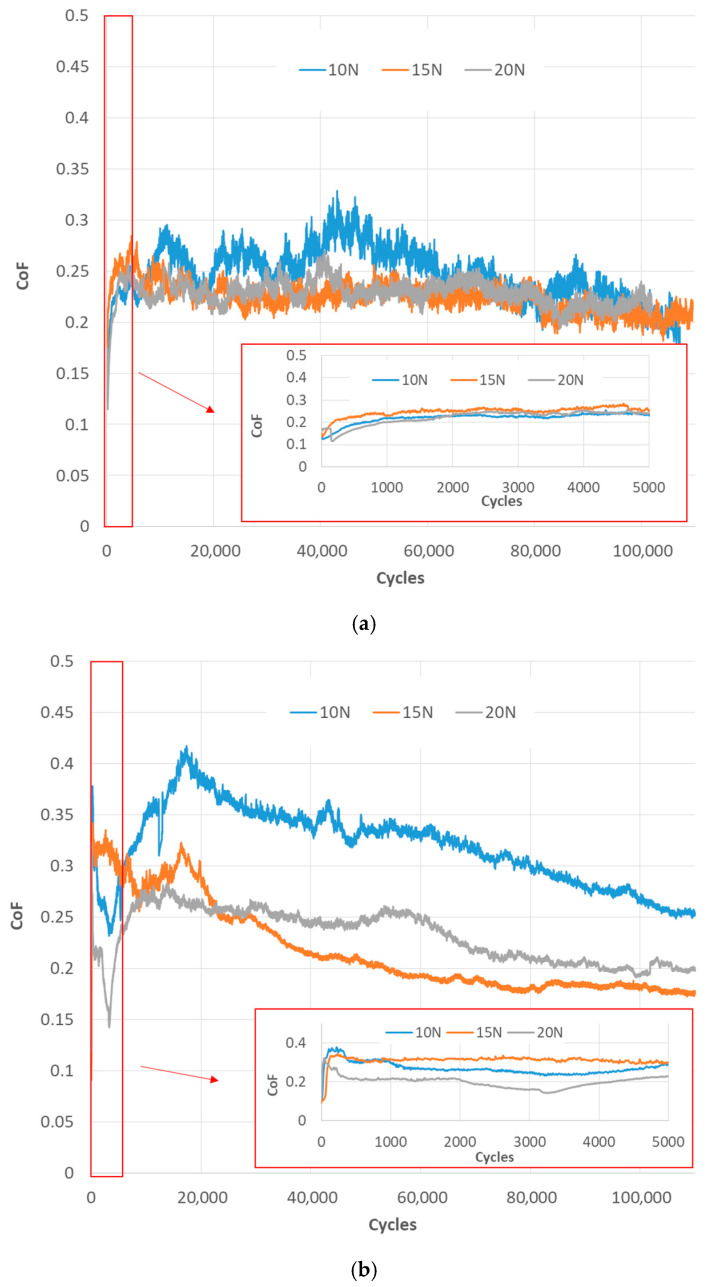
Evolution of the CoF with the number of cycles for CFRP specimens sliding against Al alloy sphere under different applied loads (10, 15, and 20 N) for (**a**) CFRPLayer specimen; (**b**) CFRPCS specimen.

Figure 6a shows the correlation between CoF and the number of cycles for the three tests performed at 10, 15, and 20 N for the CFRPLayer. Observing Figure 6a, it can be concluded that the CoF values differ significantly, indicating a lack of consistency across the tests. The 10 N applied load test exhibits the highest CoF value, while the other two tests display similar but lower CoF values. As with the previously presented results, the running-in phase is evident during the initial cycles of the test, as seen in the mini-graph that spans up to 5000 cycles. After analysing Figure 6b, which shows the relationship between the CoF and the number of cycles for the CFRPCS specimen sliding against an aluminium alloy sphere for different applied loads, it can be seen, firstly, that the data are noisier than those obtained for the titanium alloy counterbody. Secondly, as with the titanium alloy counterbody, a running-in phase was observed for the first few cycles, followed by a steady state. For these three tests at 10, 15, and 20 N applied load, the values are approximately the same and the CoF does not change much between each of them, with a coefficient of friction that ends up rounding off at 0.2 and 0.25.

**Figure 7:**
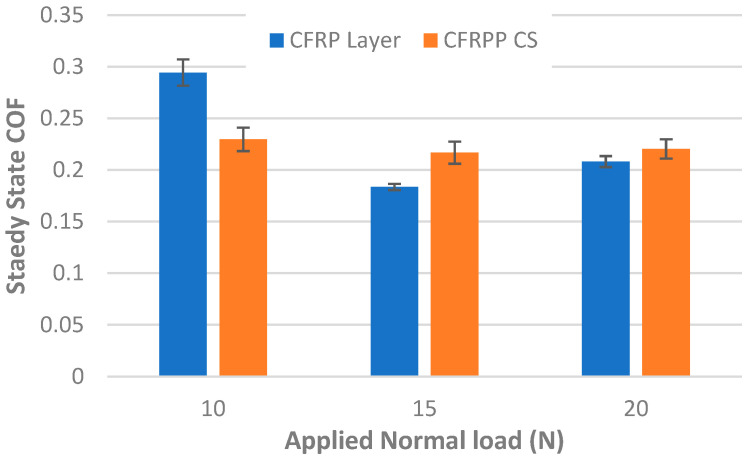
Steady-state CoF for CFRP (CFRPLayer and CFRPCS) sliding against aluminium alloy sphere under different applied loads—the steady-state region, typically from cycle 1000 to the end, was used to compute the mean CoF and standard deviation. (The coefficient of variation ranged from a minimum of 1.64% to a maximum of 4.96%).

Similarly to the other tests, the average CoF and standard deviation were calculated, and are shown in Figure 7. As can be seen for the CFRPLayer, the highest CoF belongs to the 10 N applied load test, with a value of 0.29, and the lowest to the 15 N applied load test, with a value of 0.18.

For the CFRPCS, it can be seen that the CoF value is more or less constant for all the tests, and that the highest CoF belongs to the 10 N applied load test, with a value of 0.23, and the lowest CoF to the 15 N test, with 0.22.

### 3.2. Wear Behaviour

#### 3.2.1. Wear of CFRP Sliding Against Ti Alloy as Counterbody

**Figure 8:**
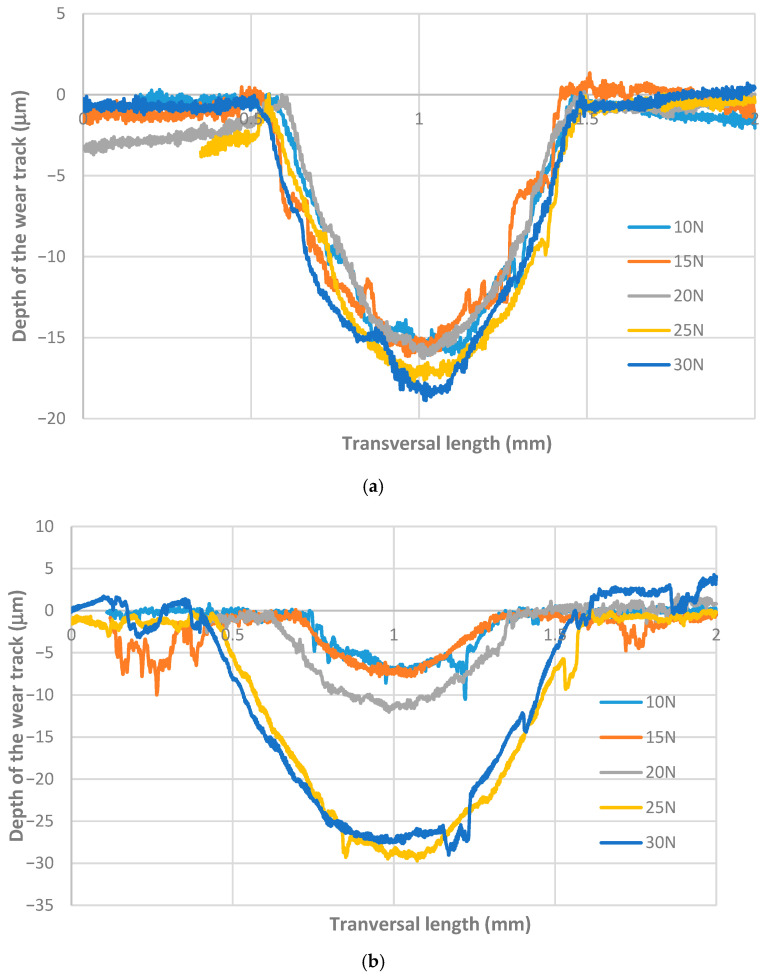
Two-dimensional profiles of the cross-section wear track from the CFRP specimens sliding against a titanium alloy sphere under varying applied loads (10, 15, 20, 25, and 30 N) for (**a**) CFRPLayer; (**b**) CFRPCS.

Figure 8a illustrates the transversal profile of the wear track for the CFRPLayer sliding against a titanium alloy sphere for varying applied loads. It can be observed that the depth of the wear track values remains relatively consistent across the five tests conducted (10, 15, 20, 25, and 30 N). However, as the load increases, the depth also exhibits a slight increase. Figure 8b presents a comparison between the depth and width of the wear track for the CFRPCS sliding against a titanium alloy sphere under different applied loads. As observed in the CFRPLayer specimens, for the same counterbody material, an increase in load results in a corresponding increase in volume. Here, it can be observed that, for the three tests with the smaller values of normal force, the wear volume was low and approximately constant. However, when considering the two tests with higher loads (25 and 30 N), it is evident that they exhibit a high degree of similarity, despite the significantly higher volumes observed in comparison to the other three tests. For both 25 N and 30 N, the wear volume increased by approximately 9% compared to the 20 N load test.

The wear volume and wear coefficient (K) were determined, and can be seen in Table 6 and Figure 8. It is evident that both the wear volume and the wear rate coefficient value are influenced by the load. For the CFRPLayer, as the load increases, a consistent increase in the wear volume and a consistent decrease in the K value are observed.

**Figure 9:**
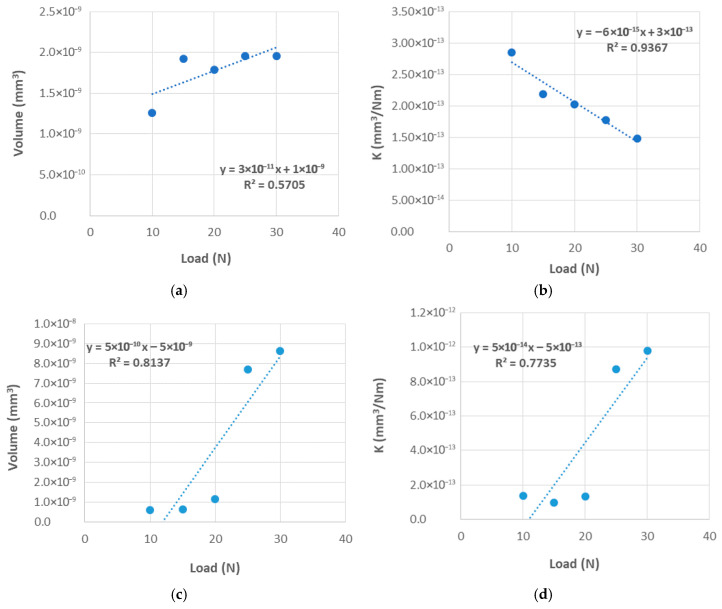
Wear volume vs. applied load for the CFRP specimens sliding against a titanium alloy sphere: (**a**) Layer, (**c**) CS. Wear coefficient vs. applied load for the CFRP specimens sliding against a titanium alloy sphere: (**b**) Layer, (**d**) CS.

As far as concerns the CFRPCS, as the load increases, a notable and consistent rise in wear volume is observed. Moreover, as illustrated in Figure 9c,d, the final two tests with loads of 25 and 30 N exhibit markedly disparate order values in comparison to the three tests performed with the lowest normal load values, showing an increase of 556% and 637%, respectively, in relation to the test performed at 20 n load. The anisotropy of the CFRP specimens makes linear regression unsuitable in these cases, as indicated by R^2^ values significantly lower than 1.

#### 3.2.2. Wear of CFRP Sliding Against Al Alloy as Counterbody

**Figure 10:**
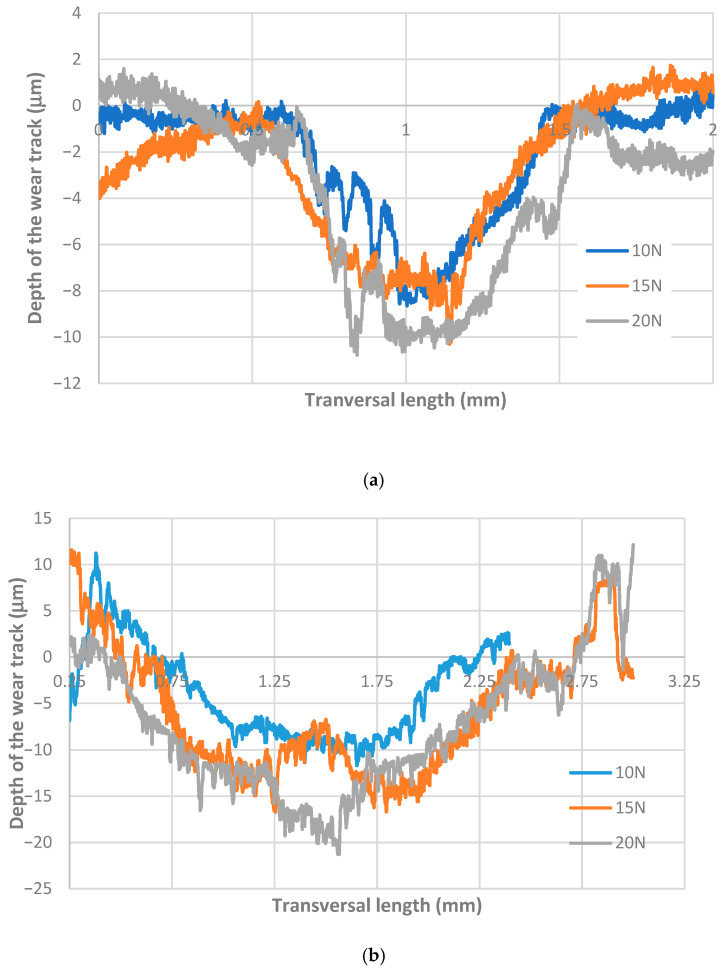
Two-dimensional profiles of the cross-section wear track of CFRP specimens sliding against an aluminium alloy sphere under varying applied loads (10, 15, and 20 N): (**a**) CFRPLayer; (**b**) CFRPCS.

Figure 10a illustrates the transversal profile of the wear track for the CFRPLayer sliding against an aluminium alloy sphere under varying applied loads. A comparable trend can be observed when the CFRPLayer is sliding against a titanium alloy sphere. The values remain relatively consistent, and, as the load increases, so does the volume. However, in this instance, the graph exhibits a more pronounced degree of noise in comparison to the data obtained from the titanium alloy experiments. This discrepancy can be attributed to the lower strength of the aluminium alloy spheres employed in the experiments in comparison to the titanium alloy spheres. The test with the lowest wear volume was that with a load of 10 N, which exhibited a wear volume of 6.53 × 10^−10^ mm^3^, while the highest wear volume was observed in the 20 N applied load test, which demonstrated a wear volume of 1.15 × 10^−9^ mm^3^.

Figure 10b illustrates the transversal wear profile of the wear track for CFRPCS sliding against an aluminium alloy sphere under varying applied loads. As observed in previous conditions, an increase in load resulted in a proportional increase in wear volume. The graph depicts three distinct tests, each representing a different load: 10, 15, and 20 N. The test with the lowest wear volume is the 10 N applied load test, which exhibited a wear volume of 1.86 × 10^−9^ mm^3^, while the test with the highest wear volume is the 20 N test, which exhibited a volume of 8.55 × 10^−9^ mm^3^. In comparison to the CFRPLayer, the wear volume is significantly higher, in a manner analogous to that observed in the titanium alloy part.

**Figure 11:**
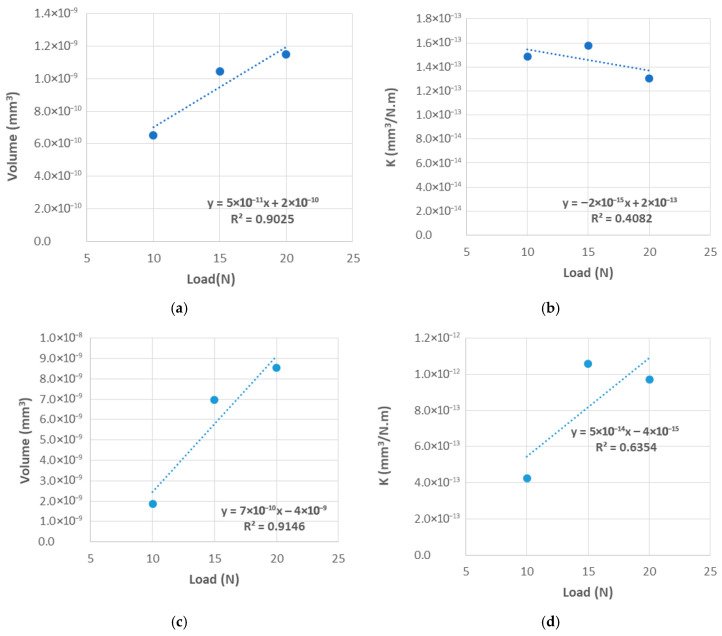
Wear volume vs. applied load for the CFRP specimens sliding against an aluminium alloy sphere: (**a**) Layer, (**c**) CS. Wear rate coefficient vs. applied load for the CFRP specimens sliding against an aluminium alloy sphere: (**b**) Layer, (**d**) CS.

As anticipated for the CFRPLayer and shown in Table 7 and Figure 11a,b, like the titanium alloy example, the volume increases as the load increases. However, when it comes to the wear rate, K, the results are not as simple. In the initial two tests (10 and 15 N), the value of K increased with the increment of the load. However, in the last test (20 N), the value decreased, and it was even lower than the initial measurement for 10 N applied load.

For the CFRPCS, as shown in Table 7 and Figure 11c,d, which present the wear volume and wear rate coefficient, it can be seen that the wear volume tends to increase with the increasing load, and that the value K has a variation comportment to the load, first increasing but later decreasing. In the analysis of the wear rate coefficient, R^2^ values well below 1 were observed, highlighting the limited predictive capability of the model.

#### 3.2.3. Wear Rate for the Ti and Al Alloy Counterbodies (20 N Applied Load)

The wear volume and the wear coefficient were also determined for the counterbody used, which in this case consisted of titanium and aluminium alloy spheres. The worn spherical cap volume was calculated using Equation (1). The wear coefficient was obtained by normalizing the worn volume with respect to the applied load (20 N) and the sliding distance (440 m). The results are presented in Table 8. From the analysis of Table 8, it can be concluded that the wear rate is significantly higher for the aluminium alloy counterbody, with the CS configuration causing the most wear. In contrast, for the titanium alloy counterbody, the wear rates are much lower than for the Al, and are of the same order of magnitude for both configurations (layer and CS).

### 3.3. Wear Mechanism

**Figure 12:**
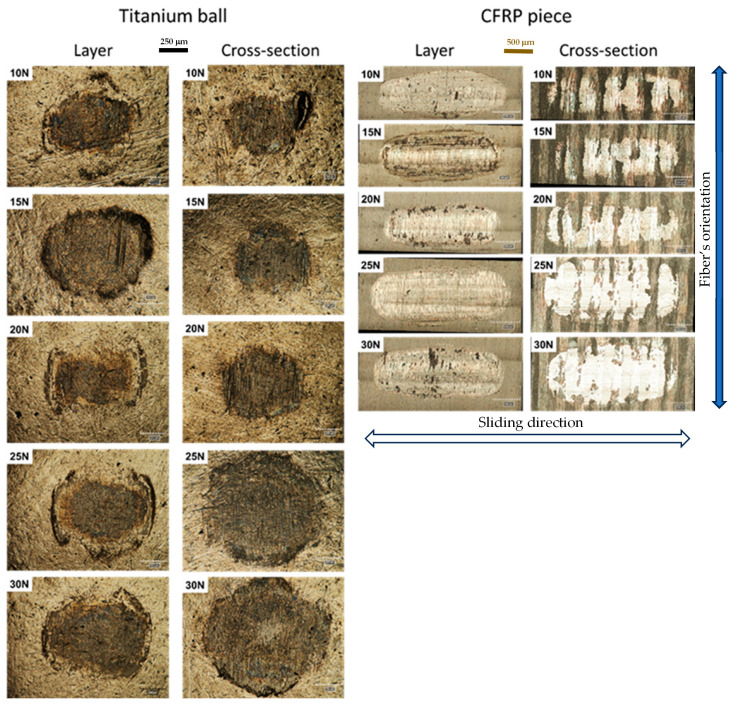
Digital micrographs (Hirox equipment) from the worn specimens: body (CFRP specimen, scale bar = 500 µm) and counterbodies (titanium alloy ball, scale bar = 250 µm) at different applied loads (10, 15, 20, 25, and 30 N).

The micrographs provided in Figure 12 clearly demonstrate the presence of wear marks on the titanium alloy balls and on the CFRP specimens in all tests, ranging from 10 to 30 N. It should be noted that all micrographs were captured at the same magnification, with the scales set to 250 μm for all titanium alloy spheres and 500 μm for all CFRP specimens. The micrographs on the left side depict the results of the five tests conducted on the layer portion of the specimen (CFRPLayer), while those on the right side illustrate the outcomes of the test on the cross-section specimen (CFRPCS). A detailed examination of the micrographs corroborates the conclusion derived from the wear volume graphs, namely that the wear value increases with the applied load following the Archard equation. This observation is corroborated by the images, which demonstrate that the wear marks become progressively larger with each increase in load. It is, however, noteworthy that the 15 N test on the surface layer is an exception to this trend, as the wear marks are significantly larger than those observed in the other tests (not a monotonically growth). The two final cross-section tests (CFRP piece, cross-section, 25 N and 30 N) exhibit the highest wear volume compared to all other tests. Additionally, there is a significant disparity in wear volume between these two tests and the first three, a phenomenon previously observed in Figure 8b. It can be observed that each micrograph of the titanium alloy sphere, with consideration given to the load and the location of the test, is comparable to the micrograph taken of the CFRP specimen. On closer examination of the micrographs of the CFRP specimen for the cross-section (CFRPCS), it becomes evident that the fibres exhibit a bidirectional orientation, with some areas displaying greater wear than others. This observation aligns with the findings of Aamir et al. [4], who attributed this phenomenon to the bidirectionality of the fibres. In the layer part, where the fibres are oriented in a single direction, it can be observed that only one direction of the fibres is in contact. However, in a few areas, particularly in the highest loads, some breaking of the fibres occurs.

CFRP tribological behaviour is highly heterogeneous, depending on whether the specimen is tested in the sliding plane coincident to the fibre’s orientation (layer) or, alternatively, with alternating fibre layers in the sliding plane and perpendicular to it (cross-section). This heterogeneity explains the non-monotonic behaviour observed in the wear evolution under the increasing applied load. Beyond the different fibre orientations, the fact that the fibres have significantly higher stiffness than the resin that acts as a binding material adds another factor of local heterogeneity.

**Figure 13:**
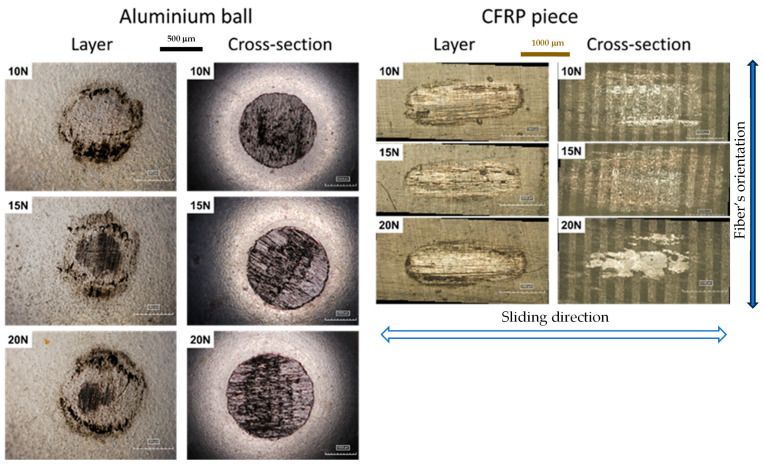
Three-dimensional digital micrographs (Hirox equipment) from the worn specimens: body (CFRP specimen, scale bar = 1000 µm) and counterbody (aluminium alloy ball, scale bar = 500 µm) at different applied loads (10, 15, and 20 N).

The micrographs presented in Figure 13 illustrate the wear marks on the aluminium alloy spheres and on the CFRP specimens across all tests, from 10 to 20 N. The images on the left side pertain to the three tests conducted on the layer part (CFRPLayer), while those on the right side correspond to the three tests on the cross-section (CFRPCS). The magnification differs between the two sides, with a scale of 500 μm on the left and 1000 μm on the right. The discrepancy in the scale is a consequence of the wear mark from the cross-section being more prominent, necessitating a reduction in magnification to facilitate comprehensive observation. A detailed examination of the micrographs reveals that, as with the titanium alloy sphere subjected to higher loads, the wear marks are more pronounced and deeper, a phenomenon that was previously elucidated through the analysis of the wear volume graphs. A comparison of the wear marks on the titanium and aluminium alloy spheres revealed that the aluminium alloy spheres exhibited significantly larger marks due to the wear. This discrepancy can be attributed to the fact that the scale on the titanium alloy photographs is smaller (250 μm) than that on the aluminium alloy photographs (500 μm or 1000 μm). The titanium alloy spheres exhibited uniform wear across regions, with no significant variation. In contrast, a notable discrepancy is evident in the aluminium alloy spheres, where the regions subjected to testing display markedly different wear patterns due to the fibre orientation effect.

**Figure 14:**
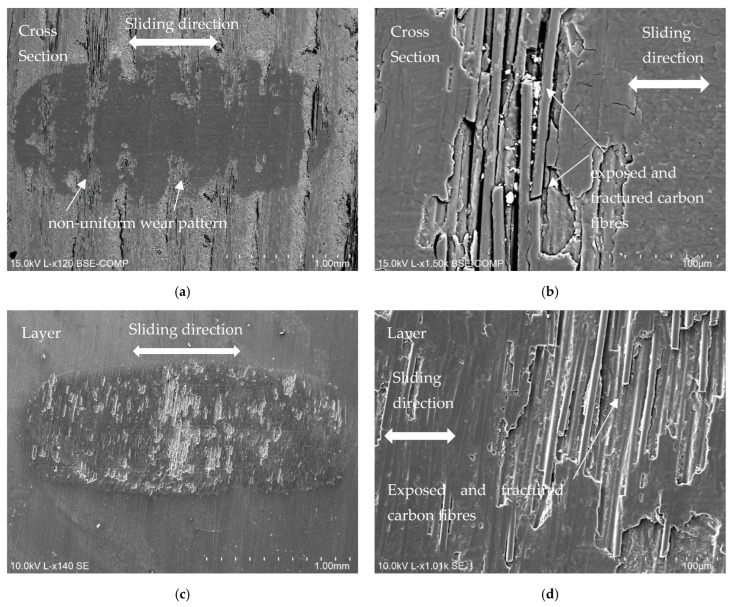
SEM micrographs from worn CFRP specimens sliding against Ti alloy counterbodies (30 N applied load): (**a**) wear track overview for cross-section configuration (scale 1 mm); (**b**) detail of worn carbon fibres for cross-section configuration (scale 100 µm); (**c**) wear track overview for layer configuration (scale 1 mm); (**d**) detail of worn carbon fibres for layer configuration (scale 100 µm).

A series of scanning electron microscopy micrographs of worn CFRP specimens sliding against a Ti alloy counterbody, either in the layer configuration or in the cross-section configuration, are displayed in Figure 14. For the cross-section configuration (Figure 14a,b), we can infer that the wear track is not homogeneous, as there are sporadic areas (vertical stripes showing a band pattern) where the wear is higher and corresponds with the fibres oriented parallel to the surface, and other areas where the wear is lower and corresponds with the carbon fibres oriented perpendicular to the surface. Figure 14b illustrates how these fibres can occasionally become broken and exposed. As stated in the introduction and discussed by Aamir et al. [4], a surface that includes multiple fibre layers oriented at different angles creates regions with varying strength and stiffness, which may justify this anisotropy in surface wear.

**Figure 15:**
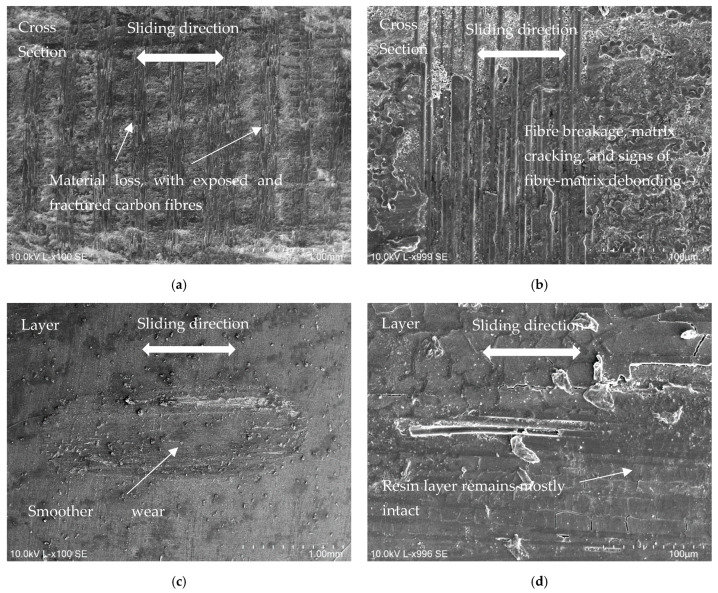
SEM micrographs from worn CFRP specimens sliding against Al alloy counterbodies (20 N applied load): (**a**) wear track overview for cross-section configuration (scale 1 mm); (**b**) detail of worn carbon fibres for cross-section configuration (scale 100 µm); (**c**) wear track overview for layer configuration (scale 1 mm); (**d**) detail of worn carbon fibres for layer configuration (scale 100 µm).

For the layer configuration, as can be seen in Figure 14c, wear is more homogenous, and this alternation of different patterns and wear resistance is not observed. However, we can also see exposed carbon fibres throughout the entire wear zone. The behaviour of the CFRP samples in relation to the Al counterbody is identical to that observed in Figure 14, with the exception that, considering that Al alloy is softer, wear is less. The worn surfaces for the cross-section configuration are depicted in Figure 15a,b, where some exposed carbon fibres can be seen following the removal of the surface resin layer and some fibre breakage. Related to the layer configuration, the surface resin layer was not entirely removed, as can be seen in Figure 15c,d, and occasionally some cracks exposing some carbon fibres can be seen.

As observed, we can conclude that the orientation of the fibres decisively influences the wear resistance. This fact had already been presented by Nguyen et al. [15], who demonstrated that different fibre orientations in CFRP affect wear, with 45° plies causing the most significant wear, while 0° plies caused the least. Additionally, the material of the counterbody and applied load also play a significant role in the wear mechanism observed.

The present study, while offering valuable insights into the tribological behaviour of CFRP against titanium and aluminium alloys, has several limitations. First, the tests were conducted under dry sliding conditions with constant sliding speed and frequency, which may not fully represent real-world aerospace environments where lubrication, temperature fluctuations, and humidity can influence performance.

## 4. Conclusions

The principal objective of the present research work was to gain a deeper understanding of the tribological behaviour of carbon fibre reinforced polymer against metallic materials such as aluminium and titanium alloys, due to their growing pair combination in composite-metal stacks (CFRP-Al and CFRP-Ti). Following the completion of the tribological tests, characterisation, demonstration of the results, and respective discussion, the following conclusions were drawn:

- The coefficient of friction (CoF) is influenced by the applied load, fibre orientation (layer vs. cross-section), and the counterbody material (Ti or Al).
- CFRP in the layer configuration showed a higher CoF than the cross-section, regardless of the metallic counterpart.
- Titanium alloy counterbodies resulted in lower CoF values compared to aluminium alloy under similar testing conditions.
- Wear volume increased proportionally with applied load for all test conditions, consistent with Archard’s wear model.
- The cross-section configuration of CFRP experienced higher wear than the layer configuration across all conditions.
- CFRP sliding against titanium alloy exhibited greater wear on the polymer surface, whereas aluminium alloy counterbodies underwent significantly higher wear, especially when paired with the CFRP cross-section.
- SEM analysis revealed that fibre orientation significantly affects wear mechanisms, with more fibre breakage and matrix removal in the cross-section configuration.
- The inherent anisotropy of CFRP contributes to heterogeneous wear behaviour, depending on fibre alignment relative to the sliding direction.
- These results provide meaningful guidance for selecting materials and orientations in composite-metal contacts, particularly in aerospace components where friction and wear performance are critical.

## Figures and Tables

**Table 1:** CFRP specifications (Reprinted from Ref. [12]).

Parameter	Specifications
Prepreg	Hexply^®^ 8552 UD AS4, unidirectional fibres
Fibre orientation	[0°, 90°]_10s_
Fibre filament count	12 K
Fibre volume fraction	57%
Resin	Hexcel^®^ Amine cured epoxy
Curing pressure	5 bar
Plate thickness	7.3 mm
Specimen’s dimensions	Squares with 20 mm sides

**Table 2:** Chemical composition of the titanium alloy (Ti6Al4V) counterbody (wt.%) (reprinted from Ref. [12]).

N	C	H	Fe	O	Al	V	Ti
0.05	0.08	0.015	0.4	0.2	5.5–6.75	3.4–4.5	bal.

**Table 3:** Chemical composition of the aluminium alloy (anodized aluminium 2024 with T6 heat treatment) counterbody (wt.%) (reprinted from Ref. [8]).

Si	Fe	Mg	Cu	Mn	Zn	Cr	Ti	Other	Al
Max. 0.5	Max. 0.5	0.3–0.9	3.8–4.9	1.2–1.8	Max. 0.25	Max. 0.1	Max. 0.15	Max. 0.15	90.7–94.7

**Table 4:** Tribological test conditions.

CFRP Fibre Orientation	Counterbody	Applied Load (N)	Sliding Time (s)	Sliding Distance (m)	Frequency (HzzHzH)	Sliding Speed (m/s)	Number of Cycles
CFRP_Layer_	Ti	10, 15, 20, 25, 30	20,000	440	5.5	0.022	110,000
	Al	10, 15, 20					
CFRP_CS_	Ti	10, 15, 20, 25, 30					
	Al	10, 15, 20					

**Table 5:** Contact radius and maximum contact pressure for different contact conditions.

Body	Counterbody	Applied Load (N)	Contact Radius, a (µm)	Maximum Contact Pressure, p_0_ (MPa)
CFRP	Ti alloy	10–30	102–147	458–662
	Al alloy	10–20	97–122	471–577

**Table 6:** Wear volume and wear coefficient for CFRP specimens sliding against a titanium alloy sphere for different normal loads (10, 15, 20, 25, and 30 N).

	Normal Load (N)	10	15	20	25	**30**
Layer	Wear volume (mm^3^)	1.26 × 10^−9^	1.92 × 10^−9^	1.79 × 10^−9^	1.95 × 10^−9^	1.95 × 10^−9^
	Wear rate coefficient K (mm^3^/N·m)	2.86 × 10^−13^	2.19 × 10^−13^	2.03 × 10^−13^	1.78 × 10^−13^	1.48 × 10^−13^
CS	Wear volume (mm^3^)	6.08 × 10^−10^	6.48 × 10^−10^	1.17 × 10^−9^	7.68 × 10^−9^	8.62 × 10^−9^
	Wear rate coefficient K (mm^3^/N·m)	1.38 × 10^−13^	9.82 × 10^−14^	1.33 × 10^−13^	8.73 × 10^−13^	9.80 × 10^−13^

**Table 7:** Wear volume and wear rate coefficient for CFRP specimens sliding against an aluminium alloy sphere for different applied loads (10, 15, and 20 N).

	Applied Load (N)	10	15	20
Layer	Wear volume (mm^3^)	6.53 × 10^−10^	1.04 × 10^−9^	1.15 × 10^−9^
	Wear rate coefficient K (mm^3^/N·m)	1.48 × 10^−13^	1.58 × 10^−13^	1.31 × 10^−13^
CS	Wear volume (mm^3^)	1.86 × 10^−9^	6.98 × 10^−9^	8.55 × 10^−9^
	Wear rate coefficient K (mm^3^/N·m)	4.24 × 10^−13^	1.06 × 10^−12^	9.71 × 10^−13^

**Table 8:** Wear volume and wear rate coefficient for the Ti and Al alloy counterbodies sliding against CFRP (20 N).

	Counterbody	Wear Volume (mm^3^)	Wear Rate Coefficient K (mm^3^/N·m)
Layer	Al	1.53 × 10^−2^	1.74 × 10^−6^
	Ti	3.71 × 10^−3^	4.21 × 10^−7^
CS	Al	6.06 × 10^−1^	6.88 × 10^−5^
	Ti	2.95 × 10^−3^	3.35 × 10^−7^

## Data Availability

The original contributions presented in this study are included in the article. Further inquiries can be directed to the corresponding author.
